# The Association Between Shear Wave Elastography-Derived Muscle Stiffness and Muscle Force/Activation in Foot and Ankle Muscles: A Systematic Review

**DOI:** 10.3390/diagnostics16121777

**Published:** 2026-06-09

**Authors:** Garrison Thornton, Scott Lucitt, Jordan Dembsky, Brody Kobler, Ian Brouk, Alexander Bombino, Caitlin Lightle, Abbis Jaffri

**Affiliations:** 1 School of Medicine, Creighton University, Omaha, NE 68178, USA; garrisonthornton@creighton.edu (G.T.); scottlucitt@creighton.edu (S.L.); jordandembsky@creighton.edu (J.D.); brodykobler@creighton.edu (B.K.); ianbrouk@creighton.edu (I.B.); alexanderbombino@creighton.edu (A.B.); caitlinlightle@creighton.edu (C.L.); 2 Department of Physical Therapy, Creighton University, Omaha, NE 68178, USA

**Keywords:** shear wave elastography, muscle stiffness, ankle, neuromuscular force, muscle activation, systematic review, ultrasound elastography, musculoskeletal

## Abstract

**Background/Objectives**: Shear wave elastography (SWE) quantifies muscle stiffness in vivo, yet its relationship with neuromuscular force and activation in ankle and foot muscles has not been systematically explored. This systematic review aimed to synthesize available evidence on the association between SWE-derived stiffness and neuromuscular force and activation outcomes in ankle and foot muscles. **Methods**: PubMed, Scopus, and CINAHL were searched through November 2025 for studies reporting SWE-derived stiffness alongside force or activation outcomes in at least one foot or ankle muscle. Methodological quality was assessed with the Downs and Black checklist, and overall certainty of evidence was evaluated using the GRADE framework. This review is registered with PROSPERO (CRD420261348340). **Results**: Twenty studies (637 participants) were included; the triceps surae was most frequently examined (16 studies). Fourteen studies reported at least one significant association between SWE stiffness and a force or activation outcome. Resting passive stiffness showed weak or absent correlations with maximal voluntary contraction torque but was consistently associated with rate of torque development at elongated muscle lengths. Active SWE stiffness tracked contraction intensity within individuals and correlated with concurrently measured joint moment. The stiffness–force relationship was modulated by joint angle, contraction state, and muscle examined. Methodological quality was fair in 17 studies and poor in three. **Conclusions**: SWE-derived stiffness is associated with neuromuscular outcomes in a context-dependent manner as follows: passive stiffness reflects explosive force capacity rather than maximal strength, while active stiffness tracks within-subject contraction intensity rather than serving as a between-subject surrogate for absolute force.

## 1. Introduction

Muscles of the ankle and foot are critical for providing strength, stability, agility, and mobility to the ankle joint. In both gait and athletic performance, these muscles greatly contribute to dynamic balance control and force transmission within the lower extremity [1]. Damage or weakness in these muscles can create ankle joint instability, increasing the likelihood of chronic pain, swelling, and sprains. For example, dysfunction of the fibular muscles can precede varus instability, whereas dysfunction in muscles of plantarflexion can precede valgus instability [2]. Proper functioning of these muscles requires efficient force transmission. In a study of wrist movements, muscle stiffness was found to be the limiting factor in force production, playing a critical role in biomechanical force generation [3]. Therefore, muscle stiffness is a key determinant of force transmission and helps to provide the mechanical impedance necessary for ankle joint function.

Given the importance of muscle stiffness in force production, quantifying stiffness can be clinically useful for joint assessment. However, conventional functional assessment methods applied to the foot and ankle have well-recognized limitations in this anatomically complex region. Surface electromyography (EMG), although widely used to characterize muscle activation, is constrained by crosstalk between adjacent muscles in the densely packed lower leg compartment and is largely unable to record from deep or intrinsic muscles. The plantar intrinsic foot muscles, including flexor digitorum brevis, quadratus plantae, abductor hallucis, and the lumbricals and interossei, lie beneath the plantar aponeurosis and thick subcutaneous tissue, rendering them effectively inaccessible to surface EMG and requiring fine-wire electrode insertion, an invasive technique impractical for routine clinical or athletic assessment [4]. Even where surface EMG is feasible, the signal reflects neural drive rather than mechanical output and cannot characterize the passive mechanical properties of resting muscle that contribute to force transmission and elastic energy storage. Isokinetic and isometric dynamometry, although reliable for quantifying joint moment, aggregate the contributions of all muscles crossing a joint and cannot isolate the behavior of individual synergists, a critical limitation in the foot and ankle, where the triceps surae, deep posterior compartment, peroneal group, and intrinsic foot muscles share overlapping mechanical actions [5]. Dynamometry is also unable to characterize passive tissue properties or detect regional mechanical variation within a single muscle. Methods specifically intended to quantify stiffness face their own constraints as follows: joint-level estimates aggregate properties of all muscles crossing a joint and cannot isolate individual muscle contributions [5]; myotonometry is influenced by adipose tissue thickness, limiting its validity in clinical settings [6]; and magnetic resonance elastography is limited by cost, equipment access, and inconsistent reliability [7]. Shear wave elastography (SWE) has emerged as a useful tool for the quantitative measurement of muscle stiffness, demonstrating both high repeatability and reproducibility with fast and non-invasive implementation [8,9].

For measuring muscle stiffness, ultrasound imaging-based SWE is usually focused on muscle belly. This is a clear difference from tendon stiffness, particularly of the Achilles tendon that has received substantial research attention because of its accessible anatomical position and well-established role in elastic energy storage and return [10]. However, the muscle belly remains the site of active force generation and the principal target of neuromuscular rehabilitation. Muscle belly stiffness reflects the combined contribution of the contractile machinery (actin–myosin cross-bridge cycling), the parallel elastic element comprising titin, extracellular matrix, and perimysium, and the active state of motor unit recruitment, properties that together determine force–length behavior, rate of force development, and resistance to imposed perturbations during locomotion [11]. These mechanical characteristics are not captured by tendon assessment, which reflects passive series elastic properties downstream of force generation. In the foot and ankle, the coordinated function of the triceps surae, tibialis anterior, and intrinsic foot muscles depends on the mechanical state of each muscle belly; force-sharing among synergists, fatigue-induced changes in contractile function, and population-specific adaptations associated with aging, athletic training, or neurological injury manifest at the muscle level [12]. Characterizing muscle belly stiffness with SWE therefore provides information that complements, rather than duplicates, existing tendon-focused research and is uniquely positioned to address mechanistic and clinical questions about neuromuscular function in this region [11].

Although publications utilizing SWE are increasing rapidly, published findings show considerable variability and are sometimes difficult to reproduce across platforms and protocols [10,13]. The present study aims to evaluate the existing literature on ankle and distal leg muscle stiffness and its relationship to muscle force and activation by SWE. By synthesizing current findings, this study seeks to clarify what is currently understood about SWE and identify patterns that may contribute to the variability of published studies.

## 2. Materials and Methods

The reporting on this study is derived from the Preferred Reporting Items for Systematic Reviews and Meta-Analyses (PRISMA) 2020 guidelines [14]. This review is registered with PROSPERO: CRD420261348340.

### 2.1. Information Sources and Search Strategy

A systematic search of PubMed, Scopus, and CINAHL was conducted in accordance with PRISMA 2020 guidelines. The completed PRISMA 2020 checklist is provided as Supplementary Table S1. An additional manual search of reference lists from included studies was performed. The date of the last search was 8 November 2025. Studies published between 2000 and 2025 were considered eligible.

**Figure 1:**
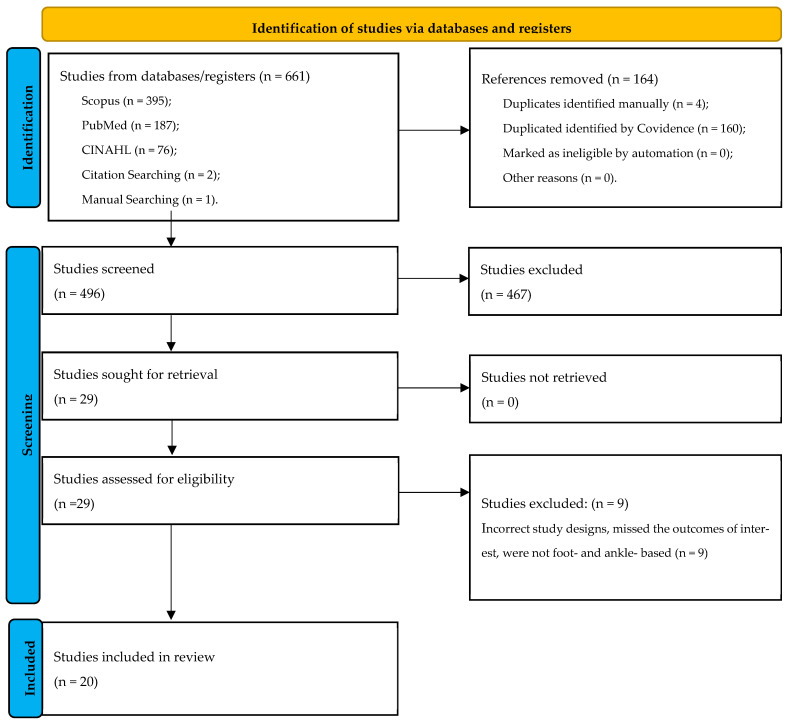
PRISMA flowchart generated by Covidence. Studies from databases/registers (*n* = 661): Scopus (*n* = 395), PubMed (*n* = 187), CINAHL (*n* = 76), citation searching (*n* = 2), manual searching (*n* = 1). After removal of 164 duplicates and screening, 20 studies met final inclusion criteria.

The PubMed search string was as follows: (“shear wave elastography” OR “shear elastic modulus” OR “shear modulus” OR SWE OR “supersonic shear imaging” OR elastography) AND (“muscle force” OR “muscle strength” OR torque OR “maximal voluntary contraction” OR MVC OR MVIC OR “isometric contraction” OR dynamometry OR electromyography OR EMG OR “muscle activation”) AND (“correlation” OR “association” OR “relationship”) AND (“lower limb” OR “lower extremity” OR leg OR ankle OR foot OR gastrocnemius OR soleus OR “tibialis anterior” OR “tibialis posterior” OR quadriceps OR hamstring* OR gluteus). Filters applied: publication date 2000–2025, English language, and human studies. The number of records identified per database is reported in the PRISMA flow diagram (Figure 1).

### 2.2. Eligibility Criteria

Original research examining the association between SWE-derived muscle stiffness and measures of muscle force and/or activation was eligible for inclusion. Studies were required to be in English, involve human subjects, and report SWE-derived stiffness outcomes for at least one foot or ankle muscle in conjunction with a force metric (e.g., dynamometry) and/or a measure of muscle activation (e.g., electromyography). Studies were excluded if they did not include any measure of muscle force or activation, if elastography outcomes were not derived using SWE, or if they focused solely on non-muscular tissues without reporting muscle stiffness. Systematic reviews and study protocols were excluded.

### 2.3. Study Selection

Title screening was conducted independently by two researchers, followed by abstract screening by two distinct reviewers. Full-text assessment was then performed independently by two additional researchers. Discrepancies were resolved through discussion and consensus at each stage.

### 2.4. Data Extraction

Data extraction was conducted by one reviewer using a standardized Excel spreadsheet. Data extracted included: (a) sample size, (b) muscles examined, (c) measurement position/joint angle, (d) contraction condition (Rest/MVC/%MVC), (e) SWE outcome (modulus/SWV), (f) force/activation outcomes (torque/RTD/EMG), and (g) correlation between SWE and force/activation.

### 2.5. Critical Appraisal of Study Quality

Four researchers independently assessed the quality of each study using the Downs and Black checklist [15]. The checklist is composed of 27 questions across the following five subsections: Reporting (10), External Validity (3), Internal Validity–Bias (7), Internal Validity–Confounding (6), and Power (1). The Power item was excluded due to its limited applicability and poor reliability in observational and biomechanical studies. The original Downs and Black inter-rater reliability analysis identified the Power item as the least reliable component of the checklist (kappa = 0.0–0.4 across raters), reflecting both the subjectivity of judging adequacy of sample size in the absence of standardized effect-size benchmarks and the infrequent reporting of a priori power calculations in observational biomechanical studies [15]. Excluding this item therefore improves the internal consistency of the appraisal without materially altering the overall quality classification, since the small sample sizes that characterize this literature are already reflected in the External Validity and Internal Validity–Confounding subdomains. Articles were classified as follows: Excellent (26–32), Good (20–25), Fair (15–19), or Poor (≤14). The overall certainty of evidence for each outcome domain was evaluated using the GRADE framework, considering risk of bias, inconsistency, indirectness, imprecision, and publication bias.

## 3. Results

### 3.1. Selected Studies

Originally, 661 articles were identified using Scopus, PubMed, CINAHL, citation, and manual searching. After the removal of 164 duplicates, 497 studies were screened. Of these, 29 met preliminary eligibility; nine were subsequently excluded based on wrong study design. Twenty studies met final inclusion criteria. The screening process is shown in Figure 1.

### 3.2. Study Characteristics

A total of 20 studies were included, comprising 637 participants across a wide range of populations, including healthy young adults, older adults, elite athletes, trained sprinters, and clinical populations such as individuals undergoing cancer treatment. Sample sizes ranged from 6 to 131 participants per study.

Across all studies, 12 distinct muscles and connective structures were examined using SWE, including the tibialis anterior, rectus femoris, vastus lateralis, vastus medialis, biceps femoris, semitendinosus, semimembranosus, sartorius, gracilis, and the triceps surae muscle group, which remained the most frequently investigated structure (16 studies). The Achilles tendon (AT) and aponeurotic structures were examined in multiple studies.

Measurement protocols were most commonly performed at the ankle in neutral, plantarflexed, and dorsiflexed positions under passive conditions and during active isometric contractions ranging from 10% to 100% of maximal voluntary contraction (MVC). Passive measurements were included in 19 studies, while 17 studies incorporated active contractions. Eight studies included explosive or dynamic tasks such as rate of torque development (RTD), drop jumps, walking, sprinting, or fatigue protocols.

SWE outcomes were reported as either shear wave velocity (SWV) (m/s) or shear modulus/Young’s modulus (kPa). Force and activation-related outcomes included joint torque or moment, RTD or rate of force development (RFD), electromyography (EMG) or motor unit behavior, ankle joint stiffness, tendon compliance, and functional performance measures. Among the 20 included studies, 14 reported statistically significant relationships between SWE-derived stiffness and force or activation outcomes.

**Figure 2:**
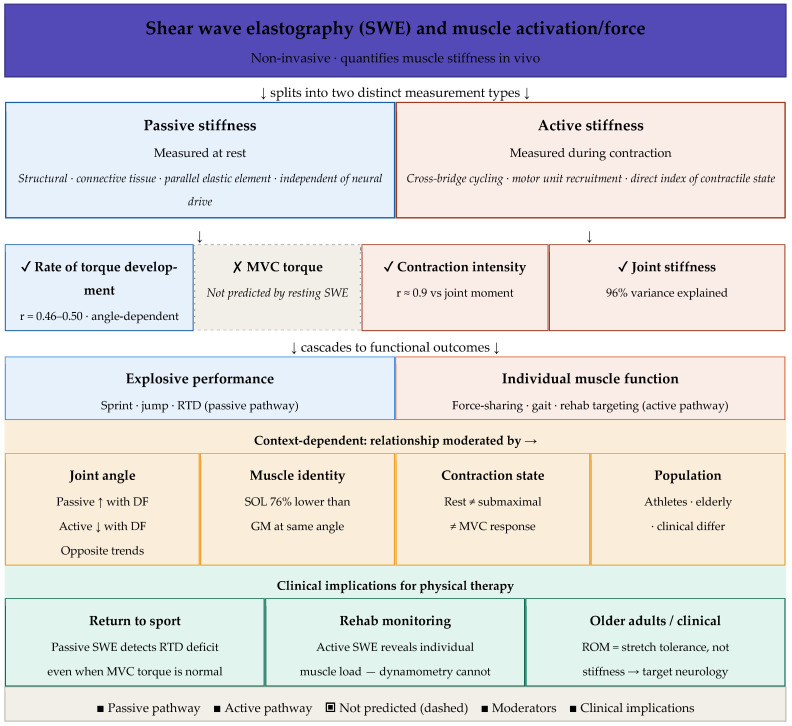
Conceptual framework illustrating the relationship between shear wave elastography (SWE)-derived muscle stiffness and neuromuscular force and activation outcomes in ankle and distal leg muscles. Passive stiffness (measured at rest) and active stiffness (measured during contraction) represent physiologically distinct signals with different neuromuscular correlates and clinical interpretations. The relationship is highly context-dependent, modulated by four key factors (joint angle, muscle identity, contraction state, and population). Arrows indicate directional pathways supported by pooled evidence. Dashed border indicates absence of a significant relationship. DF, dorsiflexion; GM, gastrocnemius medialis; MVC, maximal voluntary contraction; PEE, parallel elastic element; ROM, range of motion; RTD, rate of torque development; SOL, soleus; SWE, shear wave elastography. Background colors denote conceptual categories: blue, passive stiffness pathway; orange, active stiffness pathway; yellow, context-dependent moderators; green, clinical implications.

Resting muscle or tendon stiffness showed weak or no associations with maximal voluntary torque in six of nine studies explicitly testing this relationship. Several studies demonstrated that the stiffness–force relationship was dependent on joint angle (nine studies), contraction intensity (10 studies), muscle examined (12 studies), and population characteristics. These results are summarized in Table 1. The overall pattern of associations is synthesized in the conceptual framework presented in Figure 2.

### 3.3. Study Quality

Total Downs and Black scores ranged from 14 to 19 out of a possible 27, with no studies achieving good or excellent quality. The most frequently observed score was 17 (five studies); scores of 15 and 18 were each observed in four studies, and four studies scored 16. The lowest score (14) was observed in three studies. Based on predefined thresholds, 17 studies were classified as fair quality and three as poor quality. A summary of scores is presented in Table 2.

Reporting quality scores ranged from 6 to 9 out of 10. External validity scores ranged from 1 to 2 out of three. Internal validity–bias scores ranged from 4 to 5 out of seven, and internal validity–confounding scores ranged from 1 to 4 out of six. GRADE certainty of evidence ranged from very low to low and is summarized in Table 3.

## 4. Discussion

This systematic review synthesized evidence from 20 studies examining the association between SWE-derived muscle stiffness and neuromuscular force and/or activation in ankle and distal leg muscles. The overarching finding is that this relationship is highly context-dependent, modulated by contraction state, joint angle, the specific muscle examined, and the outcome metric of interest. Fourteen of 20 studies reported at least one statistically significant association; however, resting passive stiffness alone showed weak or absent correlations with MVC torque in six of nine studies testing this relationship directly. These findings are consistent with established biomechanical principles: passive stiffness reflects structural and connective tissue properties of the resting muscle, which are only loosely coupled to the contractile machinery that determines maximal force output. Muscle volume and physiological cross-sectional area remain the primary determinants of MVC torque, and clinicians should exercise caution before interpreting resting SWE values as a proxy for strength capacity. Taken together, these observations indicate that passive SWE-derived stiffness has limited utility as a predictive marker for comparing or estimating absolute muscle strength across different individuals, and it should not be used in isolation when the clinical or research question concerns maximal force-producing capacity.

In contrast, passive medial gastrocnemius (MG) stiffness showed a consistent and mechanistically coherent association with RTD. Ando and Suzuki [17] demonstrated significant correlations between resting MG shear modulus and normalized RTD across all tested time windows (r = 0.460–0.496), even after normalizing for MVC torque. Ando [16] further showed this relationship is joint angle-dependent, with a significant correlation emerging only at dorsiflexed positions (−15°) where the muscle is pre-loaded under passive stretch. This is consistent with the parallel elastic element (PEE) pre-loading hypothesis: at elongated muscle lengths, a stiffer PEE facilitates a steeper torque–time slope in the early phase of contraction before full motor unit recruitment occurs [18]. Yamazaki et al. [32] extended this chain of evidence in sprinters, demonstrating that passive MG shear wave speed predicted RTD, which in turn predicted 100 m sprint performance, suggesting that resting muscle stiffness conveys information about explosive capacity that conventional strength testing alone does not capture. During active contractions, SWE stiffness tracked contraction intensity with high fidelity within individual participants. Chernak et al. [21] reported linear correlations between MG shear wave velocity and normalized ankle moment of r = 0.9 across graded contraction levels within the same individuals, and Vigotsky et al. [5] demonstrated that combined plantar flexor shear wave velocities explained approximately 96% of the variance in ankle joint stiffness measured concurrently in the same participants. These findings support the use of SWE during active conditions for the following two specific within-subject applications: real-time monitoring of contraction intensity and decomposition of individual muscle contributions to joint mechanics, capabilities unavailable with dynamometry or surface EMG alone. The evidence does not, however, support the stronger claim that active SWE stiffness can serve as a between-subject surrogate for absolute muscle force. The proposal by Soldos et al. [30] that SWE provides a viable substitute for maximal isometric force testing extends beyond what cross-group correlations alone can establish, particularly given the heterogeneous pooling discussed below. Active SWE is therefore best characterized as an index of contraction intensity within a given individual and measurement session, rather than as a stand-alone estimator of absolute force across individuals.

Joint angle emerged as one of the most practically important moderators, identified in nine studies. Passive shear modulus increases with muscle elongation (dorsiflexion for plantarflexors), while active stiffness shows the opposite trend during submaximal contractions, as demonstrated by Zimmer et al. [11] across all three triceps surae heads. Keles et al. [23] further showed that SWE-derived shear modulus at MVC did not change significantly across tibialis anterior operating lengths despite large variation in joint moment, indicating that active SWE stiffness does not straightforwardly mirror the force–length relationship in all muscles. Twelve studies identified substantial muscle-specific variability as follows: the soleus displayed approximately 76% lower passive shear modulus than the gastrocnemius medialis at equivalent dorsiflexion angles, and Miyamoto and Hirata [27] cautioned that SWE shear modulus captures tissue-level intrinsic properties distinct from active stiffness estimated via B-mode ultrasound perturbation methods.

Several population-specific findings highlight the broader clinical relevance of SWE while also illustrating important interpretive caveats. In sprinters, both passive and active MG stiffness were negatively correlated with 100 m race time, and MVC torque alone was not predictive [32]. Soldos et al. [30] reported strong correlations between SWE-derived vastus lateralis elasticity and both MVC torque (r = 0.79–0.816) and RTD (r = 0.84) in a cohort spanning fast- and slow-fiber athletes, healthy non-athletes, and cancer patients. These correlations should be interpreted with caution; however, as they were calculated within pooled subgroups, the “athlete” correlation combining fast- and slow-fiber dominance participants, and the “non-athlete” correlation combining healthy controls and cancer patients, each spanning a wide range of both stiffness and force-producing capacity. Pooling heterogeneous subpopulations of this kind can inflate apparent correlation coefficients by virtue of between-group variance and does not necessarily reflect a within-individual relationship. The Soldos cohort nevertheless illustrates a constructive corollary as follows: detecting a true SWE–force relationship may require samples with sufficient inter-individual variability to overcome range restriction, a limitation likely present in the homogeneous cohorts of young, healthy participants that dominate the existing SWE literature. In older adults, Nakamura et al. [26] found that dorsiflexion ROM was associated with passive stretch tolerance but not MG shear modulus, suggesting that flexibility limitations in this population are neurologically mediated rather than structurally determined. Vincent et al. [4] demonstrated that a fatiguing isometric protocol produced parallel decreases in active MG shear modulus (−38%) and peak torque (−59%), with a significant correlation between these reductions (r = 0.6). The longitudinal training data of Ando et al. [19] are particularly informative in this regard: eight weeks of drop jump training improved reactive strength performance, but resting MG shear modulus paradoxically decreased rather than increased. This dissociation between a functional outcome and its candidate mechanical correlate is direct evidence that cross-sectional correlations between passive stiffness and explosive force capacity should not be interpreted as evidence of a causal relationship. The same passive stiffness value that correlates with RTD across individuals at a single time point does not necessarily move in the same direction as RTD when an intervention is applied within an individual over time. This distinction has important implications for the use of SWE as a serial monitoring tool in rehabilitation or athletic training, and it underscores the need for prospective longitudinal designs before passive SWE can be considered a mechanistic biomarker of explosive force capacity.

The methodological quality of included studies was rated as fair in 17 and poor in three, with no study achieving good or excellent quality. Common limitations included small sample sizes (range 6–131), low external validity, inadequate control for confounding, and a predominance of young, male, healthy convenience samples. Substantial heterogeneity in SWE outcome metrics, region-of-interest placement, joint position, contraction conditions, and ultrasound platform specifications precluded formal meta-analytic pooling. Another source of heterogeneity is cross-platform variability with different SWE systems differing in their signal processing pipelines and algorithms that add to the variance in the outcomes of different studies included in this review. Additionally, pennation angle correction, relevant for pennate muscles such as the soleus, was inconsistently applied. GRADE certainty ratings ranged from very low to low across all outcome domains (Table 3).

Finally, a critical evidence gap exists between the clinical importance of intrinsic foot muscles, emphasized in the Introduction, and the body of the SWE literature synthesized in this review, which is almost exclusively focused on extrinsic calf muscles, particularly the triceps surae. No included study examined SWE-derived stiffness in intrinsic foot muscles in relation to neuromuscular force or activation outcomes. This gap is particularly notable given recent work demonstrating that intrinsic and extrinsic foot muscles such as the abductor hallucis and tibialis posterior exhibit measurable, task-dependent stiffness changes using SWE during weight-bearing transitions, with large effect sizes (ES = −1.08 and −0.97, respectively), while the tibialis anterior and peroneal muscles do not [33]. These findings confirm that SWE is technically feasible in foot musculature and that stiffness responses are muscle- and task-specific, yet the relationship between these stiffness changes and underlying neuromuscular force or activation in intrinsic foot muscles remains entirely uncharacterized.

## 5. Future Research

Based on this systematic review, we recommend three priority directions that should guide subsequent research on SWE-derived muscle stiffness in the foot and ankle.

First, longitudinal intervention studies are required to determine whether SWE-derived stiffness is a mechanistic driver of neuromuscular performance or merely a covariate. The dissociation observed by Ando et al. [19], improved reactive strength alongside decreased resting MG shear modulus following drop jump training, shows that cross-sectional SWE–force correlations cannot be assumed to reflect causation. Pre–post designs with concurrent within-subject tracking of passive and active SWE alongside force, and muscle strength outcomes are needed to clarify whether stiffness changes precede, accompany, or lag functional change.

Second, large-scale studies in demographically diverse cohorts are required to validate SWE as a between-subject marker. The existing literature is dominated by small samples of young, healthy, predominantly male participants, producing both range restriction and limited generalizability. Future work should recruit across age, sex, training status, and pathology (e.g., chronic ankle instability, plantar fasciitis, and diabetic foot), and apply mixed-effect modeling to separate within-individual from between-group variance and so avoid the spurious pooled-group correlations described above for Soldos et al. [30].

Third, standardization of SWE acquisition and reporting protocols is a foundational prerequisite for both. Heterogeneity in platforms, region-of-interest placement, joint position, probe placement, parallel vs perpendicular projections, and contraction conditions across the included studies precluded meta-analytic pooling in the present review. Consensus guidelines for SWE of the foot and ankle musculature, analogous to existing tendon SWE guidance, would substantially improve reproducibility and accelerate clinical translation. As a starting point toward this objective, we propose a minimum reporting standard checklist (Table 4) that specifies the core acquisition and reporting elements that should be documented in future foot and ankle SWE studies.

Fourth, dedicated SWE investigation of intrinsic and extrinsic foot muscles with concurrent force and activation outcomes represents a critical and currently unaddressed research priority. Recent evidence confirms the methodological feasibility of this approach as follows: Arellano et al. [33] demonstrated that abductor hallucis and tibialis posterior stiffness increases significantly with large effect sizes during weight-bearing transitions (ES = −1.08 and −0.97, respectively), while tibialis anterior and peroneal stiffness does not, indicating distinct, muscle-specific mechanical responses to loading. Whether these stiffness changes correspond to differences in force production or neuromuscular activation remains unknown. Future studies pairing SWE with dynamometry in muscles such as the abductor hallucis, flexor digitorum brevis, and tibialis posterior are essential before the SWE–force relationships established in this review can be generalized to foot-specific conditions such as plantar fasciitis, posterior tibial tendon dysfunction, and flatfoot deformity.

## 6. Conclusions

This systematic review examined the association between SWE-derived muscle stiffness and neuromuscular force and activation outcomes across 20 studies involving 637 participants. The relationship is highly context-dependent, modulated by contraction state, joint angle, the specific muscle examined, and the outcome of interest. Resting passive stiffness consistently failed to predict maximal voluntary torque, indicating that passive SWE-derived stiffness has limited utility as a predictive marker for comparing or estimating absolute muscle strength across individuals. Passive stiffness did, however, show meaningful associations with explosive force production, particularly RTD at elongated muscle lengths where the PEE is pre-loaded; longitudinal training data caution that this cross-sectional association does not necessarily reflect a causal link, as well as serial resting SWE, should not be assumed to track changes in explosive performance within individuals. Active SWE stiffness tracked contraction intensity with high fidelity within individual participants and was capable of decomposing individual muscle contributions to joint mechanics; the evidence base supports its use as a within-subject monitoring tool rather than as a between-subject surrogate for absolute muscle force. The overall methodological quality of the evidence base was fair to poor, and substantial heterogeneity precluded definitive conclusions. Standardization of SWE measurement protocols for ankle and foot muscles, alongside prospective longitudinal designs, remains a critical prerequisite before this technology can be reliably translated into clinical or sport science practice.

## 7. Clinical Implications

The findings of this review carry several practical implications for physical therapists, orthopedic surgeons, and sports medicine clinicians. A key interpretive distinction must be established as follows: passive and active SWE stiffness reflect fundamentally different physiological phenomena. Passive stiffness, measured at rest, reflects intrinsic structural and connective tissue properties—including the extracellular matrix, titin, and the PEE—and is largely independent of neural drive. Active stiffness, measured during contraction, reflects the additional stiffness generated by cross-bridge cycling and motor unit recruitment, serving as a direct index of contractile state.

Resting passive stiffness should not be used as a surrogate for muscle strength, as it does not predict maximal voluntary torque. However, it carries meaningful information about explosive force capacity and RTD, making it a potentially useful adjunct in return-to-sport assessments where rapid force generation during sprinting, jumping, or cutting is more functionally relevant than peak force alone. A muscle that appears adequately strong on dynamometry may still exhibit reduced passive stiffness that limits its capacity for rapid elastic energy storage and release—a deficit that resting SWE can help identify. Active stiffness tracks contraction intensity with high fidelity and provides muscle-specific loading information that joint-level torque measurement cannot offer, enabling more targeted exercise prescription than surface EMG or dynamometry alone.

In older adults with restricted dorsiflexion, passive stiffness measurements are particularly informative: ROM limitations appear driven by stretch tolerance rather than elevated passive tissue stiffness, suggesting that therapeutic strategies targeting neurological tolerance are more appropriate than those aimed at mechanically reducing tissue stiffness. In patients where conventional strength testing is impractical—such as those with cancer-related muscle wasting or neurological conditions—passive SWE at rest offers a feasible non-invasive window into baseline muscle mechanical status, while serial active SWE measurements may provide sensitive indicators of neuromuscular recovery during treatment.

## Figures and Tables

**Table 1:** Summary of 20 studies included in the systematic review.

Reference	N	Muscles	Position/Angle	Contraction	SWE Outcome	Force/Activation Outcome	Correlation: SWE vs. Force/Activation
Ando, 2024 [16]	22	MG	PF (15°), Neutral (0°), DF (−15°)	Rest; MVC + explosive isometric (RTD)	SWV: 15° = 2.3 ± 0.4, 0° = 3.2 ± 0.4, −15° = 5.3 ± 0.8 m/s	MVC torque: 15° = 81, 0° = 113, −15° = 126 Nm; RTD_50_/RTD_100_/RTD_150_	Significant SWV–RTD at −15° only; no correlation at 15° or 0°
Ando and Suzuki, 2019 [17]	27	MG	Neutral (0°)	Rest; MVC + explosive isometric (RTD)	Shear modulus: 14.6 ± 3.7 kPa	MVC torque: 139.9 ± 44.6 Nm; RTD_30_–RTD_200_: 315–381% MVC/s	Shear modulus vs. normalized RTD r = 0.460–0.496; no correlation with MVC torque
Ando et al., 2021 (J Electromyogr Kinesiol) [18]	24	MG; SOL	Neutral (0°); drop jump 15 cm	Rest; MVC + explosive PF; drop jump	Shear modulus (kPa): MG = 8.3–20.4; SOL = 4.2–13.2	MVC torque: 84.9–151.5 Nm; DJ index: 0.3–2.0 m/s; jump: 6.2–38.1 cm	MG shear modulus: DJ index r = 0.414, jump r = 0.411; no correlation with RTD100/200
Ando et al., 2021 (Front Physiol) [19]	24	MG; SOL	Neutral (0°)	Rest; MVC; 8-week drop jump training	MG shear modulus (kPa): 13.5 ± 2.1→10.6 ± 2.1 (training); 12.7 ± 3.6→16.1 ± 4.3 (control)	RSI: 0.96 ± 0.42→1.48 ± 0.47 m/s; contact time: 213 ± 41→178 ± 23 ms	Stiffness changes not correlated with RTD or RSI; DJ improved despite decreased MG stiffness
Contreras-Hernandez et al., 2024 [20]	25	MG; LG; SO; AT	Neutral (0°); isometric PF at 10% and 40% MVC	Rest (AT stiffness); 10% and 40% MVC with HD-sEMG	AT stiffness: 75.95 ± 9.98 kPa; AT length: 19.89 ± 2.57 cm	Motor unit DR; COVisi; neuromechanical delay (~502 ms at 10%; ~462 ms at 40% MVC)	10% MVC: DR negatively associated with AT stiffness (41.7% variance); 40% MVC: COVisi MG+SO explained 48.7% of AT length
Chernak et al., 2013 [21]	10	MG	Max DF to max PF; knee extended vs. flexed	Passive stretch and voluntary PF up to MVC	Passive SWV: ~2.6→5.6 m/s; active: up to ~8.3 m/s	Net ankle moment (M/MVC); ICC ~0.999	r ≈ 0.8 (passive), r ≈ 0.9 (active); passive slope steeper than active
Lee et al., 2021 [22]	40	RF; BF; TA; MG	Standardized belly sites	Rest and voluntary contraction; SWE + MyotonPRO	Rest (kPa): RF~14.0, TA~21.1, MG~12.3; contraction: RF~79.8, TA~139.0, MG~79.7	Voluntary contraction; men > women	MyotonPRO vs. SWE: rest r = 0.416–0.669; contraction r = 0.398–0.594
Keles et al., 2025 [23]	14	TA	Ankle: −15° (DF), 0°, 15°, 30°, 45° (PF)	Passive; MVC; 25%, 50%, 75% MVC; SWE + EMG + moment	Passive shear modulus: ~9.67→33.05 kPa; MVC: ~122.96 kPa (stable)	Peak DF moment at 15° (~50.13 Nm); EMG varied with angle and intensity	SWE tracked contraction intensity (*p* < 0.001); discrepancy with joint-moment-derived tangent modulus
Lima et al., 2017 [24]	24	AT; MG	Prone; ankle neutral; bilateral	Rest (SWE); two 5 s MVC PF (dynamometer)	AT E: ~357–386 kPa; MG E: ~12.8–13.8 kPa; ICC: 0.821–0.986	Peak PF torque: ~130–136 Nm	Weak correlations: resting E vs. MVC torque r = 0.022 to −0.202, *p* > 0.05
Liu et al., 2020 [25]	8	VL, RF, VM, SAR, GRA, BF, SET, SEM, GM, GL	Supine/prone/sitting; gait phases	Young’s modulus; plantar pressure; 5 operators	ICC(3,1): 0.941–0.998; CV: 1.45–9.5%	Plantar pressure; GM E vs. ground reaction forces R^2^~0.936–0.987	GM E may indicate ground reaction forces; Kendall’s W ≥ 0.872
Nakamura et al., 2021 [26]	42	MG; ankle DF ROM	Seated; passive DF at 5°/s	Passive torque at DF ROM; MG shear modulus at rest	Partial correlations controlling for age/height/weight	DF ROM correlated with passive torque: men r = 0.455, women r = 0.486	DF ROM not correlated with MG shear modulus; ROM driven by stretch tolerance
Miyamoto and Hirata, 2019 [27]	17	MG	Prone; isometric PF at 10–90% MVC	Active: SWE; alpha method; B-mode US	SWE shear modulus ↑ with torque; ICCs high (SWE 0.962)	Torque and EMG increased with contraction level	SWE shear modulus not correlated with MS_Alpha or MS_US; SWE quantifies localized shear modulus independently
Ohta, 2012 [28]	6	TA	Ankle 20° PF; electrical stimulation	Single pulse vs. 2-pulse trains; IPI 5–200 ms	C2 torque > single-pulse at IPI 5–100 ms; tendinous compliance lower at short IPI	Peak torque increased at short IPI; tendon elongation decreased	Strong negative torque–compliance relationship r ≈ −0.985
Shan et al., 2021 [29]	79	Triceps surae aponeuroses; AT	Prone; ankle neutral; SSI longitudinal and transverse	Rest (aponeurosis SWV); MVC PF; 5 m walk tests	Longitudinal SWV ~6.4 ± 1.8 m/s > transverse ~3.4 ± 1.2 m/s	PF MVC: men ~127 Nm, women ~84 Nm; walk: ~1.52–2.03 m/s	Adjoining aponeurosis SWV: PF torque r = 0.23–0.34; walk speed r ≈ 0.25–0.26
Soldos et al., 2021 [30]	25	VL	Knee 90°→20°; maximal isometric	Rest; MVC; RTD (athletes only)	E vs. Mmax: athletes r = 0.79; non-athletes r = 0.816; E vs. RTDk: r = 0.84	Mmax athletes > non-athletes (+112.2%); cancer patients < healthy controls (−136%)	Strong positive correlations E vs. Mmax and RTDk; SWE proposed as surrogate for maximal isometric force
Sukanen et al., 2024 [31]	131	AT; MG, LG, SOL	Prone; AT SWE at 25° PF; TS SWE 40° PF→20° DF	Rest (AT SWV); passive stretch (TS modulus); isometric PF	AT SWV: ~7.5→10.7 m/s (distal→proximal); ICC up to 0.936	Group differences: gymnasts vs. basketball/track; no sex/sport/injury effects on most outcomes	Sport-specific adaptations in TS passive properties; acute exercise influences AT displacement
Vigotsky et al., 2020 [5]	10	MG; SOL; TA; ankle joint	Isometric PF at 0, 20, 40% MVC; knee extended vs. flexed	Active SWV; joint stiffness via perturbations; discrete muscle modeling	MG/SOL SWV explain ~96% of variance in ankle joint stiffness; SOL:MG ratio ≈ 2.83	Joint stiffness CCC up to 0.97; SWV repeatability aICC ~0.79	SWE SWV can infer muscle-specific contributions to joint stiffness
Vincent et al., 2024 [4]	12	GM; PF–tendon complex	Prone; ankle neutral/10°/20° DF; fatigue protocol: 60×4 s MVC	Pre–post fatigue: passive and active MVC	Passive: GM shear modulus ↓34% at 20° DF; Active: ↓~38%	Peak torque ↓59%; GM EMG ↓43%; RFD ↓47–62%	Active GM shear modulus vs. peak torque r ≈ 0.6; fatigue reduces both passive and active stiffness
Yamazaki et al., 2022 [32]	18	MG	Prone; ankle −15° PF to 20° DF; SWE at 0/20/50/80% MVC	Passive and active; ankle joint stiffness; explosive PF RTD	Higher passive/active MG SWV with faster 100 m time; MG SWV at 50/80% correlates with ankle joint stiffness	RTD50/RTD100 negatively correlated with 100 m time; MVC torque not related	Passive MG SWV→RTD→sprint; active MG SWV→joint stiffness→sprint; MVC torque not predictive
Zimmer et al., 2025 [11]	14	GM; GL; SOL	Prone; 30° PF, 15° PF, 0°, 15° DF	Passive; active submax (25/50/75% MVC); MVC	Passive shear modulus ↑ PF→DF; SOL ~76% lower than GM at 15° DF; Active shear modulus ↓ PF→DF	Ankle moments ↑ PF→DF; tangent modulus ↓ PF→DF; EMG ↑ with contraction	SWE captures length- and activation-dependent mechanics; distinct force-sharing across GM/GL/SOL

***Abbreviations:*** AT, Achilles tendon; BF, biceps femoris; COVisi, coherence of motor unit discharge rate; DF, dorsiflexion; DJ, drop jump; DR, motor unit discharge rate; E, Young’s modulus; EMG, electromyography; GL, gastrocnemius lateralis; GM, gastrocnemius medialis; GRA, gracilis; HD-sEMG, high-density surface EMG; ICC, intraclass correlation coefficient; IPI, inter-pulse interval; LG, lateral gastrocnemius; MG, medial gastrocnemius; MVC, maximal voluntary contraction; PF, plantar flexion; RF, rectus femoris; RFD, rate of force development; ROM, range of motion; RSI, reactive strength index; SAR, sartorius; SEM, semimembranosus; SET, semitendinosus; SO/SOL, soleus; SWE, shear wave elastography; SWV, shear wave velocity; TA, tibialis anterior; VL, vastus lateralis; VM, vastus medialis. Numbers in brackets correspond to reference list. ↑ indicates an increase in the reported measure; ↓ indicates a decrease in the reported measure.

**Table 2:** Downs and Black methodological quality scores for included studies.

Study (Author, Year)	Reporting (0–10)	External Validity (0–3)	Internal Validity–Bias (0–7)	Internal Validity–Confounding (0–6)	Total Score (0–31)	Quality Rating
Ando, 2024 [16]	8	1	5	1	**15**	Fair
Ando et al., 2021 (J Electromyogr Kinesiol) [18]	8	1	5	3	**17**	Fair
Ando et al., 2021 (Front Physiol) [19]	8	1	5	3	**17**	Fair
Ando and Suzuki, 2019 [17]	8	1	4	3	**16**	Fair
Chernak et al., 2013 [21]	8	1	5	3	**17**	Fair
Contreras-Hernandez et al., 2024 [20]	7	1	4	3	**15**	Fair
Keles et al., 2025 [23]	9	1	4	4	**18**	Fair
Liu et al., 2020 [25]	7	1	4	3	**15**	Fair
Lee et al., 2021 [22]	7	1	4	3	**15**	Fair
Lima et al., 2017 [24]	6	1	5	2	**14**	Poor
Miyamoto and Hirata, 2019 [27]	9	1	4	3	**17**	Fair
Nakamura et al., 2021 [26]	9	1	4	2	**16**	Fair
Ohta, 2012 [28]	8	1	4	1	**14**	Poor
Shan et al., 2021 [29]	9	2	4	3	**18**	Fair
Soldos et al., 2021 [30]	8	1	5	2	**16**	Fair
Sukanen et al., 2024 [31]	8	2	4	3	**17**	Fair
Vigotsky et al., 2020 [5]	8	1	4	1	**14**	Poor
Vincent et al., 2024 [4]	9	1	4	2	**16**	Fair
Yamazaki et al., 2022 [32]	9	2	4	3	**18**	Fair
Zimmer et al., 2025 [11]	9	2	4	3	**18**	Fair

**Quality Rating Key:** Excellent = 26–32; Good = 20–25; Fair = 15–19; Poor: 14. Numbers in brackets correspond to reference list citations.

**Table 3:** GRADE summary of findings: certainty of evidence for the association between SWE-derived muscle stiffness and neuromuscular force/activation outcomes in ankle and foot muscles.

Outcome	Studies (*n*)	Participants (*n*)	Risk of Bias	Inconsistency	Indirectness	Imprecision	Publication Bias	Certainty of Evidence
1. Passive SWE stiffness vs. MVC torque	9	~310	Serious ↓↓ (all fair–poor; low external validity)	Serious ↓↓ (weak/no correlation in 6/9 studies; inconsistent direction)	Not serious	Serious ↓↓ (sample sizes 6–131; no pooled estimates)	Undetected (likely present)	⊕◯◯◯ VERY LOW Passive stiffness does not reliably predict maximal strength
2. Passive SWE stiffness vs. RTD/RFD	6	~200	Serious ↓↓ (all fair quality; limited confound control)	Not serious (consistent positive associations at elongated lengths)	Not serious	Serious ↓↓ (small samples; angle-dependency limits generalizability)	Undetected	⊕⊕◯◯ LOW Consistent association at elongated muscle lengths; PEE pre-loading mechanism plausible
3. Active SWE stiffness vs. contraction intensity	10	~350	Serious ↓↓ (fair quality; operator dependence; varied ROI placement)	Not serious (strong, consistent dose–response across studies)	Not serious	Moderate ↓ (varied contraction conditions; no pooled estimates)	Undetected	⊕⊕◯◯ LOW Active SWE tracks contraction intensity reliably; decomposes individual muscle contributions
4. SWE stiffness vs. EMG/motor unit activation	6	~180	Serious ↓↓ (fair–poor quality; small samples; EMG inconsistent)	Serious ↓↓ (inconsistent associations; varies by muscle, contraction, EMG method)	Not serious	Serious ↓↓ (very small samples; wide variability in effect size)	Undetected	⊕◯◯◯ VERY LOW Relationship between SWE and neural activation poorly characterized
5. SWE stiffness vs. functional performance	6	~210	Serious ↓↓ (fair quality; cross-sectional; selection bias)	Moderate ↓ (positive associations in sprint/jump but paradoxical decrease post-training)	Not serious	Serious ↓↓ (small samples; lack of longitudinal data)	Undetected	⊕◯◯◯ VERY LOW Promising associations in sprinters; caution warranted for rehabilitation monitoring
6. SWE stiffness vs. fatigue-related torque decline	2	~50	Serious ↓↓ (fair quality; very small samples; single-session designs)	Not serious (parallel decreases consistently observed)	Not serious	Very serious ↓↓↓ (only 2 studies; very small *n*)	Undetected	⊕◯◯◯ VERY LOW Active SWE sensitive to fatigue-induced torque loss; insufficient evidence for clinical application

**Certainty ratings:** ⊕⊕⊕⊕ High | ⊕⊕⊕◯ Moderate | ⊕⊕◯◯ Low | ⊕◯◯◯ Very Low. Ratings begin at Low certainty (observational studies) per GRADE conventions. SWE, shear wave elastography; MVC, maximal voluntary contraction; RTD, rate of torque development; RFD, rate of force development; EMG, electromyography; RSI, reactive strength index; ROI, region of interest; PEE, parallel elastic element. Arrows (↓ and ↓↓ and ↓↓↓) are not self-evident symbols, they represent **GRADE downgrading notation**, which has a specific technical meaning that readers unfamiliar with the GRADE framework would not automatically understand.

**Table 4:** Proposed minimum reporting standard checklist for shear wave elastography studies of the foot and ankle musculature.

Domain	Element	Minimum Reporting Requirement
Device and platform	Manufacturer/model	Report manufacturer, model, software version, and SWE technology type (e.g., 2D-SWE, point SWE, and supersonic shear imaging).
	Transducer	Probe type (linear/curvilinear), frequency range (MHz), and footprint dimensions.
	Acoustic settings	Imaging depth, sample box dimensions, and push-pulse settings where accessible.
Subject preparation	Position	Body position (prone, supine, and seated) with documented knee and hip angles. Important to mention if imaging was performed in weight bearing or non-weight bearing positions.
	Rest and warm-up	Pre-measurement rest period and any standardized warm-up or familiarization trials.
Ankle configuration	Joint angle	Ankle angle reported in degrees relative to neutral (0°), positive for dorsiflexion; measurement method specified (goniometer, fixed footplate).
	Stabilization	Fixation method (dynamometer footplate, custom jig) and any ankle strapping.
Muscle identification	Anatomical landmarks	Probe placement defined relative to bony or palpable landmarks (e.g., 30% of leg length distal to the popliteal crease for medial gastrocnemius).
	Imaging plane	Longitudinal alignment along the dominant fiber direction; transverse imaging used only with explicit rationale.
Region of interest	ROI size and depth	ROI dimensions in mm or % of muscle cross-section and depth from skin.
	Sampling	Minimum of three acquisitions per condition; within-condition reliability (ICC or coefficient of variation) reported.
Contraction condition	Activation state	Rest, passive stretch, or active contraction; for active contractions, %MVC and force-feedback method specified.
	Concurrent recordings	Whether EMG and joint moment were recorded concurrently with SWE and at matched time points.
Outcome metric	Reporting units	Shear wave velocity (m/s) and shear modulus (kPa) both reported where possible to enable cross-study comparison.
	Statistical summary	Mean, standard deviation, and individual data points or ranges.
Operator	Experience	Operator experience level (years, prior training).
	Reliability	Intra- and inter-operator reliability (ICC, SEM, and MDC) for the specific protocol.

Abbreviations: EMG, electromyography; ICC, intraclass correlation coefficient; MDC, minimal detectable change; MVC, maximal voluntary contraction; ROI, region of interest; SEM, standard error of measurement; SWE, shear wave elastography.

## Data Availability

No new data were created or analyzed in this study. Data sharing is not applicable to this article.

## Supplementary Materials

The following supporting information can be downloaded at https://www.mdpi.com/article/10.3390/diagnostics16121777/s1, Table S1: PRISMA 2020 checklist for the systematic review.

## Abbreviations

The following abbreviations are used in this manuscript:

AT	Achilles tendon
DF	Dorsiflexion
DJ	Drop jump
EMG	Electromyography
GM	Gastrocnemius medialis
GL	Gastrocnemius lateralis
GRADE	Grading of Recommendations, Assessment, Development, and Evaluation
MG	Medial gastrocnemius
MVC	Maximal voluntary contraction
PEE	Parallel elastic element
PF	Plantarflexion
PRISMA	Preferred Reporting Items for Systematic Reviews and Meta-Analyses
RFD	Rate of force development
ROM	Range of motion
RSI	Reactive strength index
RTD	Rate of torque development
SOL	Soleus
SWE	Shear wave elastography
SWV	Shear wave velocity
TA	Tibialis anterior
VL	Vastus lateralis
