# Supplementary material for: The Association Between Shear Wave Elastography-Derived Muscle Stiffness and Muscle Force/Activation in Foot and Ankle Muscles: A Systematic Review
Source: Diagnostics (Basel). 2026 Jun 9;16(12):1777. doi: 10.3390/diagnostics16121777 (PMC13298605; doi:10.3390/diagnostics16121777)
Supplement: Supplementary file 1 [file diagnostics-16-01777-s001.zip › diagnostics-4323550-supplementary.pdf]

PRISMA 2020 Checklist

Section and Topic	Item #	Checklist item	
			Title page — manuscript title reads: “The Association Between Shear Wave Elastography-Derived Muscle Stiffness and Muscle Force/Activation in Foot and Ankle Muscles: A Systematic Review”; the design (systematic review) is identified in the title
**ABSTRACT**			p.1 — structured abstract present with Background/Objectives, Methods, Results, and Conclusions subsections; reports the number of included studies (n=20) and participants (n=637), databases searched (PubMed, Scopus, CINAHL), risk-of-bias tool (Downs and Black), certainty-of-evidence framework (GRADE), and PROSPERO registration (CRD420261348340)
**INTRODUCTION**			Section 1 (Introduction), paragraphs 2–4 — limitations of myotonometry, MRE, and joint-level measures described; SWE rationale and evidence gaps established
Objectives	4	Provide an explicit statement of the objective(s) or question(s) the review addresses.	Section 1, final paragraph — “The present study aims to evaluate the existing literature on ankle and distal leg muscle stiffness and its relationship to muscle force and activation by

PRISMA 2020 Checklist

Section and Topic	Item #	Checklist item	
			Title page — manuscript title reads: “The Association Between Shear Wave Elastography-Derived Muscle Stiffness and Muscle Force/Activation in Foot and Ankle Muscles: A Systematic Review”; the design (systematic review) is identified in the title
			SWE”
**METHODS**			Section 2.2 — inclusion: English language, human subjects, SWE-derived stiffness for ≥1 ankle/foot muscle paired with force/activation measure; exclusion: no force/activation measure, non-SWE elastography, systematic reviews, non-ankle/foot or non-muscular tissues
Information sources	6	Specify all databases, registers, websites, organisations, reference lists and other sources searched or consulted to identify studies. Specify the date when each source was last searched or consulted.	Section 2.1 — PubMed, Scopus, and CINAHL searched; manual reference list search performed; date of last search: 8 November 2025; publication years 2000–2025
Search strategy	7	Present the full search strategies for all databases, registers and websites, including any filters and limits used.	Section 2.1 — full PubMed search string presented verbatim across four conceptual domains; field-tag adaptations noted per platform; filters: 2000–2025, English, human studies
Selection process	8	Specify the methods used to decide whether a study met the inclusion criteria of the review, including how many reviewers screened each record and each report retrieved, whether they worked independently, and if applicable, details of automation tools used in the process.	Section 2.3 — title screening by two independent researchers; abstract screening by two distinct

Section and Topic	Item #	Checklist item	
			Title page — manuscript title reads: “The Association Between Shear Wave Elastography-Derived Muscle Stiffness and Muscle Force/Activation in Foot and Ankle Muscles: A Systematic Review”; the design (systematic review) is identified in the title
			reviewers; full-text assessment by two additional independent researchers; discrepancies resolved by discussion and consensus
Data collection process	9	Specify the methods used to collect data from reports, including how many reviewers collected data from each report, whether they worked independently, any processes for obtaining or confirming data from study investigators, and if applicable, details of automation tools used in the process.	Section 2.4 / Author Contributions — primary data extraction performed by one reviewer (G.T.) using a standardised Excel spreadsheet; extracted data independently verified by six additional reviewers (S.L., J.D., B.K., I.B., A.B., C.L.); discrepancies resolved by discussion and consensus; no contact with original study investigators and no automation tools used
Data items	10a	List and define all outcomes for which data were sought. Specify whether all results that were compatible with each outcome domain in each study were sought (e.g. for all measures, time points, analyses), and if not, the methods used to decide which results to collect.	Section 2.4 — outcomes listed: joint torque/moment, RTD/RFD, EMG/motor unit measures, ankle joint stiffness, functional performance; all compatible results per study extracted
	10b	List and define all other variables for which data were sought (e.g. participant and intervention characteristics, funding sources). Describe any assumptions made about any missing or unclear information.	Section 2.4 — additional variables extracted:

PRISMA 2020 Checklist

Section and Topic	Item #	Checklist item	Title page — manuscript title reads: "The Association Between Shear Wave Elastography-Derived Muscle Stiffness and Muscle Force/Activation in Foot and Ankle Muscles: A Systematic Review"; the design (systematic review) is identified in the title
			sample size, muscles examined, measurement position/joint angle, contraction condition (Rest/MVC/%MVC), SWE outcome metric (modulus vs. SWV)
Study risk of bias assessment	11	Specify the methods used to assess risk of bias in the included studies, including details of the tool(s) used, how many reviewers assessed each study and whether they worked independently, and if applicable, details of automation tools used in the process.	Section 2.5 — Downs and Black checklist used; four independent researchers assessed all 20 studies; power item excluded with rationale; scores compared and consensus reached; quality categories defined
Effect measures	12	Specify for each outcome the effect measure(s) (e.g. risk ratio, mean difference) used in the synthesis or presentation of results.	Section 2.4 / Section 3.2 — correlation coefficients (r, r^2) used as primary association measures; specific r values reported per study in Results and Table 1
Synthesis methods	13a	Describe the processes used to decide which studies were eligible for each synthesis (e.g. tabulating the study intervention characteristics and comparing against the planned groups for each synthesis (item #5)).	Section 3.2 — studies grouped by outcome domain (MVC torque, RTD/RFD, EMG, functional performance, fatigue) based on reported outcomes; qualitative synthesis used due to clinical and methodological heterogeneity

PRISMA 2020 Checklist

Section and Topic	Item #	Checklist item	Title page — manuscript title reads: “The Association Between Shear Wave Elastography-Derived Muscle Stiffness and Muscle Force/Activation in Foot and Ankle Muscles: A Systematic Review”; the design (systematic review) is identified in the title
	13b	Describe any methods required to prepare the data for presentation or synthesis, such as handling of missing summary statistics, or data conversions.	Section 2.4 — SWE outcomes reported as published (shear wave velocity m/s or shear modulus kPa); no unit conversions performed; data presented as reported by authors
	13c	Describe any methods used to tabulate or visually display results of individual studies and syntheses.	Section 3.2 / Table 1 / Figure 2 — results tabulated in structured evidence table (Table 1) and synthesised in conceptual framework figure (Figure 2)
	13d	Describe any methods used to synthesize results and provide a rationale for the choice(s). If meta-analysis was performed, describe the model(s), method(s) to identify the presence and extent of statistical heterogeneity, and software package(s) used.	Section 3.2 and Section 4 — narrative synthesis used; meta-analysis not performed due to substantial heterogeneity in SWE metrics, joint positions, contraction conditions, and ultrasound platforms; rationale stated explicitly in Discussion
	13e	Describe any methods used to explore possible causes of heterogeneity among study results (e.g. subgroup analysis, meta-regression).	Section 3.2 / Section 4 — sources of heterogeneity explored narratively: joint angle (9 studies), contraction state (10 studies), muscle examined (12 studies), population characteristics, SWE

PRISMA

PRISMA 2020 Checklist

Section and Topic	Item #	Checklist item	
			Title page — manuscript title reads: "The Association Between Shear Wave Elastography-Derived Muscle Stiffness and Muscle Force/Activation in Foot and Ankle Muscles: A Systematic Review"; the design (systematic review) is identified in the title
			platform; formal subgroup analysis not planned or conducted
	13f	Describe any sensitivity analyses conducted to assess robustness of the synthesized results.	Not applicable — no meta-analysis performed; narrative synthesis only; formal sensitivity analysis not conducted
Reporting bias assessment	14	Describe any methods used to assess risk of bias due to missing results in a synthesis (arising from reporting biases).	Section 4 (methodological quality paragraph) — publication bias acknowledged narratively; positive results more likely published in this emerging field; formal funnel plot not feasible without pooled data
Certainty assessment	15	Describe any methods used to assess certainty (or confidence) in the body of evidence for an outcome.	Section 2.5 / Table 3 — GRADE framework applied across six outcome domains; certainty rated from very low to low; summary of findings presented in Table 3
**RESULTS**			Section 3.1 / Figure 1 — 661 records identified; 164 duplicates removed; 497 screened; 29 met preliminary eligibility; 9 excluded (wrong study design); 20 included; PRISMA flow diagram Figure 1

PRISMA 2020 Checklist

Section and Topic	Item #	Checklist item	Title page — manuscript title reads: "The Association Between Shear Wave Elastography-Derived Muscle Stiffness and Muscle Force/Activation in Foot and Ankle Muscles: A Systematic Review"; the design (systematic review) is identified in the title
	16b	Cite studies that might appear to meet the inclusion criteria, but which were excluded, and explain why they were excluded.	Section 3.1 / Figure 1 (PRISMA flow diagram) — 9 studies were excluded at the full-text eligibility stage based on wrong study design; individual citations of excluded studies are reported in the Covidence-generated PRISMA flow diagram (Figure 1) rather than the main text
Study characteristics	17	Cite each included study and present its characteristics.	Section 3.2 / Table 1 — all 20 included studies cited; characteristics: sample size, population, muscles, joint positions, contraction conditions, SWE outcomes, force/activation outcomes reported
Risk of bias in studies	18	Present assessments of risk of bias for each included study.	Section 3.3 / Table 2 — Downs and Black scores for all 20 studies; domain scores (reporting, external validity, internal validity—bias, internal validity—confounding) presented; quality categories assigned
Results of individual studies	19	For all outcomes, present, for each study: (a) summary statistics for each group (where appropriate) and (b) an effect estimate and its precision (e.g. confidence/credible interval), ideally using structured tables or plots.	Table 1 — individual study results with correlation coefficients and significance;

Section and Topic	Item #	Checklist item	Title page — manuscript title reads: “The Association Between Shear Wave Elastography-Derived Muscle Stiffness and Muscle Force/Activation in Foot and Ankle Muscles: A Systematic Review”; the design (systematic review) is identified in the title
			confidence intervals not universally available as studies reported varying statistics; noted as a limitation in Section 4
Results of syntheses	20a	For each synthesis, briefly summarise the characteristics and risk of bias among contributing studies.	Section 3.2 / Section 3.3 — characteristics and quality of contributing studies summarised narratively per outcome domain; Downs and Black scores cross-referenced
	20b	Present results of all statistical syntheses conducted. If meta-analysis was done, present for each the summary estimate and its precision (e.g. confidence/credible interval) and measures of statistical heterogeneity. If comparing groups, describe the direction of the effect.	Not applicable — no meta-analysis performed; qualitative narrative synthesis used; direction of effects described in Section 3.2 and Section 4
	20c	Present results of all investigations of possible causes of heterogeneity among study results.	Section 4 — causes of heterogeneity explored narratively: joint angle dependency (9 studies), contraction state (10 studies), muscle-specificity (12 studies), population characteristics, SWE platform and metric variability
	20d	Present results of all sensitivity analyses conducted to assess the robustness of the synthesized results.	Not applicable — no meta-analysis or formal sensitivity analysis conducted

Section and Topic	Item #	Checklist item	Title page — manuscript title reads: "The Association Between Shear Wave Elastography-Derived Muscle Stiffness and Muscle Force/Activation in Foot and Ankle Muscles: A Systematic Review"; the design (systematic review) is identified in the title
Reporting biases	21	Present assessments of risk of bias due to missing results (arising from reporting biases) for each synthesis assessed.	Section 4 (methodological quality paragraph) — publication bias acknowledged as likely; positive-result publication tendency in emerging field noted; quantitative assessment not feasible without pooled data
Certainty of evidence	22	Present assessments of certainty (or confidence) in the body of evidence for each outcome assessed.	Table 3 (GRADE Summary of Findings) — certainty rated for 6 outcome domains: passive stiffness vs. MVC torque (Very Low); passive stiffness vs. RTD (Low); active stiffness vs. contraction intensity (Low); SWE vs. EMG (Very Low); SWE vs. functional performance (Very Low); active stiffness vs. fatigue (Very Low)
**DISCUSSION**			Section 4 — findings interpreted against biomechanical theory (PEE hypothesis, force-length relationship); results contextualised against prior SWE literature in other muscle groups
	23b	Discuss any limitations of the evidence included in the review.	Section 4

PRISMA 2020 Checklist

Section and Topic	Item #	Checklist item	
			Title page — manuscript title reads: “The Association Between Shear Wave Elastography-Derived Muscle Stiffness and Muscle Force/Activation in Foot and Ankle Muscles: A Systematic Review”; the design (systematic review) is identified in the title
			(methodological quality paragraph) and Table 3 (GRADE) — limitations of the included evidence: small sample sizes (range 6–131; predominantly <30), low external validity (homogeneous young, healthy, predominantly male convenience samples), inadequate confounding control, substantial heterogeneity in SWE outcome metrics, ROI placement, joint position, contraction conditions, and ultrasound platform/signal-processing pipelines; inconsistent pennation-angle correction in pennate muscles (e.g., soleus); cross-sectional designs predominate; overall GRADE certainty ranged from Very Low to Low across all six outcome domains
	23c	Discuss any limitations of the review processes used.	Section 4 — limitations of review processes: substantial clinical and methodological heterogeneity precluded meta-analytic pooling; effect sizes could not be

Section and Topic	Item #	Checklist item	Title page — manuscript title reads: "The Association Between Shear Wave Elastography-Derived Muscle Stiffness and Muscle Force/Activation in Foot and Ankle Muscles: A Systematic Review"; the design (systematic review) is identified in the title
			quantitatively synthesised; primary data extraction performed by one reviewer (although independently verified by six additional reviewers); search restricted to English-language, human studies indexed in PubMed/Scopus/CINAHL with no formal grey-literature search beyond manual reference-list checking; potential for publication bias in this emerging field could not be assessed quantitatively (no funnel plot)
	23d	Discuss implications of the results for practice, policy, and future research.	Section 5 (Future Research) and Section 6 (Clinical Implications) — clinical implications: passive vs. active SWE reflect distinct physiology; resting passive stiffness should not be used as a strength surrogate but informs explosive force capacity (RTD); active stiffness reliable for within-subject monitoring of contraction intensity; applications in older

PRISMA

PRISMA 2020 Checklist

Section and Topic	Item #	Checklist item	Title page — manuscript title reads: “The Association Between Shear Wave Elastography-Derived Muscle Stiffness and Muscle Force/Activation in Foot and Ankle Muscles: A Systematic Review”; the design (systematic review) is identified in the title
			adults, cancer/neurological populations, and return-to-sport assessment outlined; future research priorities: (i) longitudinal intervention studies, (ii) demographically diverse cohorts across age, sex, training status, and pathology (chronic ankle instability, plantar fasciitis, diabetic foot), and (iii) standardisation of SWE acquisition and reporting (proposed minimum reporting checklist, Table 4)
**OTHER INFORMATION**			Sections 1 (Abstract) and 2 — this review is prospectively registered with PROSPERO (registration number CRD420261348340)
	24b	Indicate where the review protocol can be accessed, or state that a protocol was not prepared.	Protocol prospectively registered and publicly accessible via the PROSPERO database (registration number CRD420261348340); no separate protocol publication
	24c	Describe and explain any amendments to information provided at registration or in the protocol.	No amendments were made to the registered protocol (PROSPERO

PRISMA

PRISMA 2020 Checklist

Section and Topic	Item #	Checklist item	Title page — manuscript title reads: “The Association Between Shear Wave Elastography-Derived Muscle Stiffness and Muscle Force/Activation in Foot and Ankle Muscles: A Systematic Review”; the design (systematic review) is identified in the title
			CRD420261348340) during conduct of the review
Support	25	Describe sources of financial or non-financial support for the review, and the role of the funders or sponsors in the review.	Funding section — “This research received no external funding.” No funders or sponsors had any role in the review
Competing interests	26	Declare any competing interests of review authors.	Conflicts of Interest section — “The authors declare no conflicts of interest.”
Availability of data, code and other materials	27	Report which of the following are publicly available and where they can be found: template data collection forms; data extracted from included studies; data used for all analyses; analytic code; any other materials used in the review.	Data Availability Statement — “Data are available on request from the corresponding author.” The extracted data are summarised in Tables 1–3; no separate code or analytic scripts were generated (narrative synthesis); template data collection form available on request

From: Page MJ, McKenzie JE, Bossuyt PM, Boutron I, Hoffmann TC, Mulrow CD, et al. The PRISMA 2020 statement: an updated guideline for reporting systematic reviews. BMJ 2021;372:n71. doi: 10.1136/bmj.n71.
This work is licensed under CC BY 4.0. To view a copy of this license, visit https://creativecommons.org/licenses/by/4.0/
